# Comparison of predictors for early and late mortality in adults commencing HIV antiretroviral therapy in Zimbabwe: a retrospective cohort study

**DOI:** 10.1186/s12981-022-00445-4

**Published:** 2022-05-28

**Authors:** Bradley W. Byers, Douglas Drak, Tinei Shamu, Cleophas Chimbetete, Rumbi Dahwa, David M. Gracey

**Affiliations:** 1 Central Clinical School, Faculty of Medicine, The University of Sydney, Sydney, NSW Australia; 2 Royal North Shore Hospital, St Leonards, NSW Australia; 3 Department of Renal Medicine, Royal Prince Alfred Hospital, Camperdown, NSW Australia; 4 Newlands Clinic, Newlands, Harare, Zimbabwe; 5 Institute of Social and Preventive Medicine, University of Bern, Bern, Switzerland; 6 Graduate School of Health Sciences, University of Bern, Bern, Switzerland; 7 Faculty of Medicine and Health Sciences, University of Zimbabwe, Harare, Zimbabwe; 8 Central Clinical School, Room 5214, Level 2, Medical Foundation Building 92–95 Parramatta Road, 2050 Sydney, Australia

**Keywords:** HIV, Anti-retroviral therapy, Mortality, CD4 count, Zimbabwe, Sub-Saharan Africa

## Abstract

**Background:**

People living with HIV (PLWHIV) commencing antiretroviral therapy (ART) in sub-Saharan Africa experience significant mortality within the first year. Previously, identified risk factors for mortality may be biased towards these patients, as compared to those who experience late mortality.

**Aim:**

To compare risk factors for early and late mortality in PLWHIV commencing ART.

**Methods:**

A retrospective cohort study of ART-naïve patients aged ≥ 18 years from an outpatient HIV clinic in Zimbabwe. Data were collected between January 2010 and January 2019. Predictors for early (≤ 1 year) and late mortality (> 1 year) were determined by multivariable cox proportional hazards analyses, with patients censored at 1 year and landmark analysis after 1 year, respectively.

**Results:**

Three thousand and thirty-nine PLWHIV were included in the analysis. Over a median follow-up of 4.6 years (IQR 2.5–6.9), there was a mortality rate of 8.8%, with 50.4% of deaths occurring within 1 year. Predictors of early mortality included CD4 count < 50 cells/µL (HR 1.84, 95% CI 1.24–2.72, p < 0.01), WHO Stage III (HR 2.05, 95% CI 1.28–3.27, p < 0.01) or IV (HR 2.83, 95% CI 1.67–4.81, p < 0.01), and eGFR < 90 mL/min/1.73 m^2^ (HR 2.48, 95% CI 1.56–3.96, p < 0.01). Other than age (p < 0.01), only proteinuria (HR 2.12, 95% CI 1.12–4.01, p = 0.02) and diabetes mellitus (HR 3.51, 95% CI 1.32–9.32, p = 0.01) were associated with increased risk of late mortality.

**Conclusions:**

Traditional markers of mortality risk in patients commencing ART appear to be limited to early mortality. Proteinuria and diabetes are some of the few predictors of late mortality, and should be incorporated into routine screening of patients commencing ART.

## Introduction

In 2020, there were approximately 25 million people living with HIV (PLWHIV) in sub-Saharan Africa, accounting for more than two thirds of the global burden of HIV [1]. People living with HIV who commence antiretroviral therapy (ART) in the region have been shown to have mortality rates of 5–41%, across follow-up periods of up to 60 months, with the majority of deaths occurring within the first year [2, 3]. Similar high early mortality is not observed in resource-rich settings [4].

In cohorts of PLWHIV from sub-Saharan Africa, low baseline CD4 count, eGFR, and WHO disease stage have been identified as risk factors for mortality [2, 3, 5, 6]. What remains unclear, however, is whether these factors differ between those who succumb to early mortality (within the first year after commencing ART) and those who die later. Those who are at risk of late mortality may not be identified by established predictors, while potentially being more amenable to clinical intervention. There are limited available data at present to evaluate these assertions, however. Here we compare predictors of early and late mortality, in a large cohort of PLWHIV commencing ART, from an outpatient HIV clinic in Zimbabwe.

## Methods

 This was a retrospective cohort study of PLWHIV who commenced ART at the Newlands Clinic in Harare, Zimbabwe, a non-government organisation funded by charitable donors which provides free HIV care. Patients who attended the clinic between January 2010 and January 2019 were assessed for eligibility. Inclusion criteria included: age ≥ 18 years, ART-naïve, and having baseline (prior to the initiation of ART treatment) CD4 counts and serum creatinine measurements available. Ethical approval for this study was granted by the Medical Research Council of Zimbabwe (MRCZ/E/258). All research activities were conducted in accordance with the Declaration of Helsinki.

### Data collection and analysis

Data were collected from patient electronic medical records. Variables of interest included baseline age, sex, BMI, WHO stage, serum creatinine and CD4 count. Additionally, a documented histories of proteinuria, diabetes mellitus and hypertension at baseline were recorded. Baseline serum creatinine was used to calculate eGFR utilizing the CKD-EPI formula [7]. Renal impairment was defined as a baseline eGFR < 90 mL/min/1.73 m^2^, severe immunodeficiency as a CD4 count < 50 cells/µL, and proteinuria as urine dipstick protein of ≥1 +.

### Statistical analysis

Patients were grouped into those who experienced no mortality, early mortality (within one year of starting ART) and those who experienced late mortality (over one year of ART). Comparisons between groups were by unpaired t-tests, one-way ANOVA, and chi-square, as appropriate. Games Howell post-hoc test was applied to ANOVA where homogeneity of variance was not satisfied. To compare predictors for early and late mortality, two multivariable cox proportional hazards analyses were conducted. For early mortality risk factors, a regression was conducted where patients were censored after one year. For late mortality, a landmark regression analysis was performed, limited to patients with over one year of survival data. Potential predictors were included a priori and included sex, age, BMI, proteinuria, diabetes mellitus, hypertension, WHO stage, renal impairment, and severe immunodeficiency. Data are presented as median (IQR) or mean ± SD where appropriate.

## Results

Between January 2010 and January 2019, 3039 patients at the Newlands Clinic met inclusion criteria for this study. The median age of the cohort was 36 years (IQR 30–43), with the majority of the cohort being female (62.1%). Over a median follow-up time of 4.6 years (IQR 2.5–6.9), there was a mortality rate of 8.8%. Approximately half of the patients who died (132/266, 50.4%) did so within one year of commencing ART.

Baseline characteristics by mortality status are presented in Table 1. Surviving patients had a higher BMI (24.6 ± 12.5 vs. 22.0 ± 5.4 kg/m^2^; p < 0.01) and a greater baseline CD4 count (290.2 ± 222.5 vs. 165.1 ± 172.9 cells/µL; p < 0.01) than those who died in the follow-up period, and a greater proportion of surviving patients were a lower WHO stage (p < 0.01). Amongst those who died, those who did so within the first year had a lower BMI (21.2 ± 4.7 vs. 23.4 ± 6.3 kg/m^2^; p = 0.02), baseline CD4 count (141.9 ± 161.3 vs. 188.9 ± 181.5 cells/µL; p = 0.03), and prevalence of hypertension (7% vs. 17%; p = 0.01) compared to those who experienced late mortality.

**Table 1:** Baseline characteristics of all adult patients

	No mortality (n = 2773)	All mortality (n = 266)	p value	Early mortality (n = 134)	Late mortality (n = 132)	p value
Age (years)	36.0 (29.0, 42.0)	39.0 (32.0, 48.0)	< 0.01	39.0 (29.0, 42.0)	39.0 (32.0, 47.8)	0.78
Sex (female—n)	1753 (63%)	135 (51%)	< 0.01	69 (51%)	66 (50%)	0.81
BMI (kg/m^2^)	24.6 ± 12.5	22.0 ± 5.4	< 0.01	21.2 ± 4.7	23.4 ± 6.3	**0.02**
WHO stage (n)	Stage I: 1351 (51%) Stage II: 559 (21%) Stage III: 512 (19%) Stage IV: 241 (9%)	Stage I: 41 (17%) Stage II: 45 (18%) Stage III: 82 (33%) Stage IV: 79 (32%)	< 0.01	Stage I: 19 (15%) Stage II: 20 (16%) Stage III: 43 (34%) Stage IV: 45 (35%)	Stage I: 21 (18%) Stage II: 26 (22%) Stage III: 38 (32%) Stage IV: 34 (28%)	0.48
eGFR (mL/min/1.73 m^2^)	130.5 ± 28.6	124.3 ± 29.0	< 0.01	123.0 ± 32.2	125.7 ± 25.5	0.46
eGFR < 90 mL/min/1.73 m^2^ (n)	187 (7%)	35 (13%)	< 0.01	23 (17%)	12 (9%)	0.05
CD4 count (cells/µL)	290.2 ± 222.5	165.1 ± 172.9	< 0.01	141.9 ± 161.3	188.9 ± 181.5	0.03
CD4 count	286 (10%)	83 (31%)	< 0.01	52 (39%)	31 (23%)	0.01
Proteinuria (n)	265 (9%)	79 (30%)	< 0.01	44 (36%)	35 (28%)	0.20
Diabetes Mellitus (n)	62 (2%)	11 (4%)	0.05	4 (3%)	7 (5%)	0.34
Hypertension (n)	462 (17%)	31 (12%)	0.03	9 (7%)	22 (17%)	0.01

Values are expressed as number (%), median (IQR), or mean ± SD

* BMI* body mass index; *WHO* World Health Organization; *eGFR* estimated glomerular filtration rate

Multivariable proportional hazards analyses of potential predictors of early and late mortality are presented in Table 2. A reduced eGFR (< 90 mL/min/1.73 m^2^) was associated with more than doubling of early mortality risk (HR 2.48, 95% CI 1.56–3.96, p < 0.01), but was not associated with increased late mortality risk (p = 0.59). In contrast, proteinuria was associated with a similarly increased risk of both early mortality (HR 2.29, 95% CI 1.59–3.31, p < 0.01) and late mortality risk (HR 2.12, 95% CI 1.12–4.01, p = 0.02). Hypertension and BMI were associated with reduced early mortality (HR 0.56, 95% CI 0.33–0.94, p = 0.03 and HR 0.95, 95% CI 0.92, 0.99, p = 0.02 respectively), but not late mortality (p = 0.42 and p = 0.80, respectively). Diabetes mellitus predicted mortality in both the early (HR 2.33, 95% CI 1.10–4.91, p = 0.03) and the late mortality group (HR 3.51, 95% CI 1.32–9.32, p = 0.01). For early mortality, WHO stage III (HR 2.05, 95% CI 1.28–3.27, p < 0.01) and stage IV (HR 2.83, 95% CI 1.67–4.81, p < 0.01) were significant predictors, but were not for late mortality (p = 0.08 and p = 0.40, respectively). A CD4 count < 50 cells/µL was also associated with early (HR 1.84, 95% CI 1.24–2.72, p < 0.01) but not late mortality (p = 0.71).

**Table 2:** Multivariable Cox proportional hazards assessment of baseline predictors of mortality in adult patients undergoing ART

	Early mortality		Late mortality	
	HR (95% CI)	p value	HR (95% CI)	p value
Sex (female)	1.16 (0.82, 1.63)	0.41	0.98 (0.56,1.72)	0.94
Age	1.04 (1.03, 1.06)	< 0.01	1.05 (1.02, 1.07)	< 0.01
BMI	0.95 (0.92, 0.99)	0.02	0.99 (0.94, 1.05)	0.80
Proteinuria	2.29 (1.59, 3.31)	< 0.01	2.12 (1.12, 4.01)	0.02
Diabetes mellitus	2.33 (1.10, 4.91)	0.03	3.51 (1.32, 9.32)	0.01
Hypertension	0.56 (0.33, 0.94)	0.03	0.75 (0.37, 1.51)	0.42
WHO stage I (reference)	–	–	–	–
WHO stage II	1.52 (0.93, 2.49)	0.10	1.01 (0.48, 2.12)	0.98
WHO stage III	2.05 (1.28, 3.27)	< 0.01	1.82 (0.93, 3.59)	0.08
WHO stage IV	2.83 (1.67, 4.81)	< 0.01	1.49 (0.59,3.81)	0.40
eGFR < 90 mL/min/1.73 m^2^	2.48 (1.56, 3.96)	< 0.01	1.29 (0.51, 3.3)	0.59
CD4 count < 50 cells/µL	1.84 (1.24, 2.72)	< 0.01	0.85 (0.37, 1.98)	0.71

*h* hazard ratio; *CI* confidence interval; *BMI* body mass index; *eGFR* estimated glomerular filtration rate

## Discussion

Our study compared risk factors for early and late mortality in PLWHIV commencing ART in sub-Saharan Africa. Our findings suggest that previously established risk factors for mortality, including low CD4 count, WHO stage III or IV, and reduced renal function, may not identify patients at risk of death after more than one year of ART.

WHO stage III and IV are well established predictors of mortality [2, 8–10]. While these stages were associated with more than a doubling of early mortality risk in our study, we did not find an increased risk after 1 year of ART. A similar trend was noted by Moore et al. [11] in a cohort from Uganda comparing patients who died before or after 3 months of ART. There, WHO stage III or IV was associated with a nine-fold increased risk of early mortality, but only a three-fold increase in mortality after 3 months. This apparent waning of the predictive value of WHO stage may relate in part to immune reconstitution inflammatory syndrome (IRIS), which is more common in patients with more advanced immunodeficiency on commencement of ART [12–14]. Haddow et al. [15] reported a cumulative incidence of IRIS of 22.9% in their cohort of adult PLWHIV initiating ART in South Africa, with IRIS accounting for nearly one quarter of total clinical events and deaths within the first six months of treatment. This suggests a meaningful contribution of IRIS to early mortality. However, other research in sub-Saharan Africa has shown that IRIS did not largely contribute to mortality in adult PLWHIV undergoing ART treatment—one study in Uganda demonstrated that 7% of total HIV-related deaths within the first three months of ART were related to IRIS [16], whereas another study in Johannesburg observed that among IRIS patients, there was a 4.5% IRIS-related mortality rate within 6 months of starting ART [17]. Therefore, the contribution of IRIS in the present study is unclear. Whether late mortality risk could be predicted by restaging patients after a period of ART remains a possibility, however, and is an important avenue for further research.

A baseline eGFR < 90 mL/min/1.73 m^2^ was associated with a 2.5-fold increase in mortality risk of early, but not late mortality. Reports of other cohorts from the region have also identified eGFR as a significant risk factor for mortality, with lower levels of renal function being associated with greater mortality risk [5, 6]. We were unable to determine if more severe renal impairment was associated with a late mortality risk as few patients in our cohort had a baseline eGFR < 60 mL/min/1.73 m^2^. As our study used a single measure of creatinine to determine baseline renal function, we were also unable to differentiate patients with acute kidney injury from those with chronic kidney disease.

The presence of proteinuria at baseline was associated with a doubling of both early and late mortality risk. As with serum creatinine, baseline proteinuria status was assessed by a single measure. It may be that dipstick proteinuria is less affected than serum creatinine by factors such as hydration status or muscle wasting and so provides a more reliable assessment than a single measure of serum creatinine. Proteinuria status is also important to identify patients with potential HIV-associated nephropathy, which presents as proteinuria with rapidly progressive renal failure [18].

The apparent protective effect of hypertension in the early mortality group was likely artefactual, a consequence of normotensive and hypotensive patients being grouped together. Hypotension has previously been shown to increase mortality risk in a cohort of PLWHIV from Kenya, with increasing severity of hypotension linked to increasing mortality risk. However, this association was not observed in the subset of patients with advanced HIV disease [19]. While hypertension was not associated with late mortality risk in our cohort, this may have been due to a median follow-up time of only 2.9 years. However, deleterious changes from hypertension have been shown to occur within this timeframe. In previously published data from this same cohort, hypertension predicted renal function loss over a period of only six months [20]. These findings suggest the clinical importance of identifying those with hypotension, as this may contribute to elevated early mortality risk.

A higher body mass index was a protective factor for early mortality, as noted in other cohorts of PLWHIV commencing ART in sub-Saharan Africa [2, 3]. A lower baseline BMI has also been associated with increased mortality within the first 3 months [21–23]. Zachariah et al. [22] observed a linear trend between increased mortality risk within the first 3 months and reductions in BMI below 18.5–16 kg/m^2^, with a marked increase in mortality risk once BMI falls below 16 kg/m^2^. This is likely a result of those with lower BMI representing a complex interaction between undernourishment in addition to HIV-associated wasting, as sub-Saharan Africa is disproportionately affected by both food scarcity and HIV infection [24]. Together, these factors increase disease progression, likely contributing to the exaggerated early mortality observed in PLWHIV with low BMI initiating ART in sub-Saharan Africa [21–23]. One study from Zambia indicated a significant prevalence of wasting in a cohort of people with severe HIV initiating ART, with 34% of the cohort having a BMI of < 18.5 kg/m^2^ at baseline [25]. The increased early mortality observed in sub-Saharan PLWHIV initiating ART is believed to be a culmination of immune dysfunction secondary to malnutrition, metabolic derangements resulting from an increased basal metabolic rate and/or selective skeletal muscle atrophy, as well as increased burden of opportunistic infections [26].

The main limitation of this study is that it examined ambulatory patients at a single clinic in Zimbabwe, and so may not be generalizable to other populations in sub-Saharan Africa. Reports of other cohorts have found similar protective benefits of a higher BMI, as well as increased risk of mortality associated with reduced eGFR, CD4 count, and WHO Stage III and IV [2, 3, 27]. These studies also had similar baseline BMI, mean CD4 count and eGFR compared to the present study. Therefore, it is likely that the present findings are generalizable to the larger sub-Saharan African PLWHIV population.

## Conclusions

Traditional risk factors for mortality including CD4 count, WHO stage, and renal impairment did not identify patients at increased mortality risk after one year from ART commencement in the present study. Late mortality risk was increased by the presence of proteinuria and having diabetes mellitus, however, and screening for these risk factors should form part of routine clinical assessment.

## Data Availability

The datasets used in the current study are available from the corresponding author on reasonable request.

## Abbreviations

ART: Antiretroviral therapy
BMI: Body mass index
eGFR: Estimated glomerular filtration rate
HIV: Human immunodeficiency virus
HR: Hazard ratio
IQR: Interquartile range
IRIS: Immune reconstitution inflammatory syndrome
PLWHIV: People living with HIV
WHO: World Health Organization
